# The influence of edema on the bisoprolol blood concentration after bisoprolol dermal patch application: A case-control study

**DOI:** 10.1097/MD.0000000000027354

**Published:** 2021-09-24

**Authors:** Yuji Takahashi, Tomohiro Sonoo, Hidehiko Nakano, Hiromu Naraba, Hideki Hashimoto, Kensuke Nakamura

**Affiliations:** Department of Emergency and Critical Care Medicine, Hitachi General Hospital, 2-1-1, Jonan-cho, Hitachi, Ibaraki, Japan.

**Keywords:** beta blocker, bisoprolol, blood concentration, dermal patch, edema

## Abstract

**Background:**

Beta-blocking is important for critically ill patients. Although some patients are required to continue taking beta-blockers after they no longer need critical care, some of these patients have impaired swallowing abilities. Bisoprolol dermal patches have recently been introduced and appear to be a good alternative to oral bisoprolol tablets. However, it is still unclear whether the pharmacodynamics of such patches are affected by edema in patients who have experienced critical care. This study aimed to clarify the effects of systemic edema on beta-blocker absorption from dermal patches in critically ill patients.

**Method:**

Patients who exhibited tachycardia and impaired swallowing function after critical care were included in this study. They were assigned to either the edema group (n = 6) or no edema group (n = 6) depending on the presence/absence of edema in the lower extremities. A bisoprolol dermal patch was pasted onto each subject, and the blood bisoprolol concentration was checked at 8 timepoints over the next 24 hours. The area under the serum concentration time curve, maximum concentration observed (C_max_), and time of maximum concentration observed were also examined.

**Result:**

The mean blood bisoprolol concentrations of the 2 groups were not significantly different at 2, 4, 6, 8, 10, 12, 16, or 24 hours after the patch application. The area under the serum concentration time curve and maximum concentration observed were not different between the groups. The mean heart rates of the 2 groups were not significantly different at 6, 12, or 24 hours after the patch application (Student *t* test, *P* = .0588, *P* = .1080, and *P* = .2322, respectively).

**Conclusion:**

In this study, the blood concentration of bisoprolol and its heart rate-reducing effects after bisoprolol dermal patch application might not be affected by systemic edema in the lower extremities.

## 1 Introduction

Patients in the ICU often have tachycardic arrhythmia that needs controlling because structural or functional heart diseases are common among such patients. Such tachyarrhythmia requires prompt control, as it can lead to circulatory instability. Recently, it has been reported that short-acting beta-blocker use in critically ill septic patients resulted in better survival.^[1]^ Since the publication of these findings, esmolol and landiolol, which are short-acting beta-blockers, have been frequently used in the ICU setting.

In the ICU, many patients receive intravenous (IV) short-acting beta-receptor antagonists, and these drugs are subsequently switched to oral formulations. However, especially in patients who were once critically ill, swallowing function can be drastically impaired, which can result in difficulties with oral drug administration. Recently, bisoprolol dermal patches, Bisono Tape (Toa Eiyo Ltd., Tokyo, Japan), has become commercially available in Japan. A previous study revealed that 4-mg and 8-mg bisoprolol patches are not inferior to 2.5 mg and 5 mg oral bisoprolol, respectively, in terms of the ability of these drugs to reduce patients’ heart rates (HR) and blood pressure (BP).^[2]^ Also, a previous study introduced a strategy involving critically ill patients being switched from IV landiolol to bisoprolol dermal patches.^[3]^ However, it must be considered that critically ill patients often have moderate to severe edema, which might reduce the effects of dermal patch medications. As far as we searched, no previous studies have investigated the effects of edema on the absorption of dermal bisoprolol patch. The purpose of this study was to clarify the effects of moderate to severe edema on bisoprolol absorption from dermal patches in critically ill patients.

## 2 Materials and method

**Figure 1:**
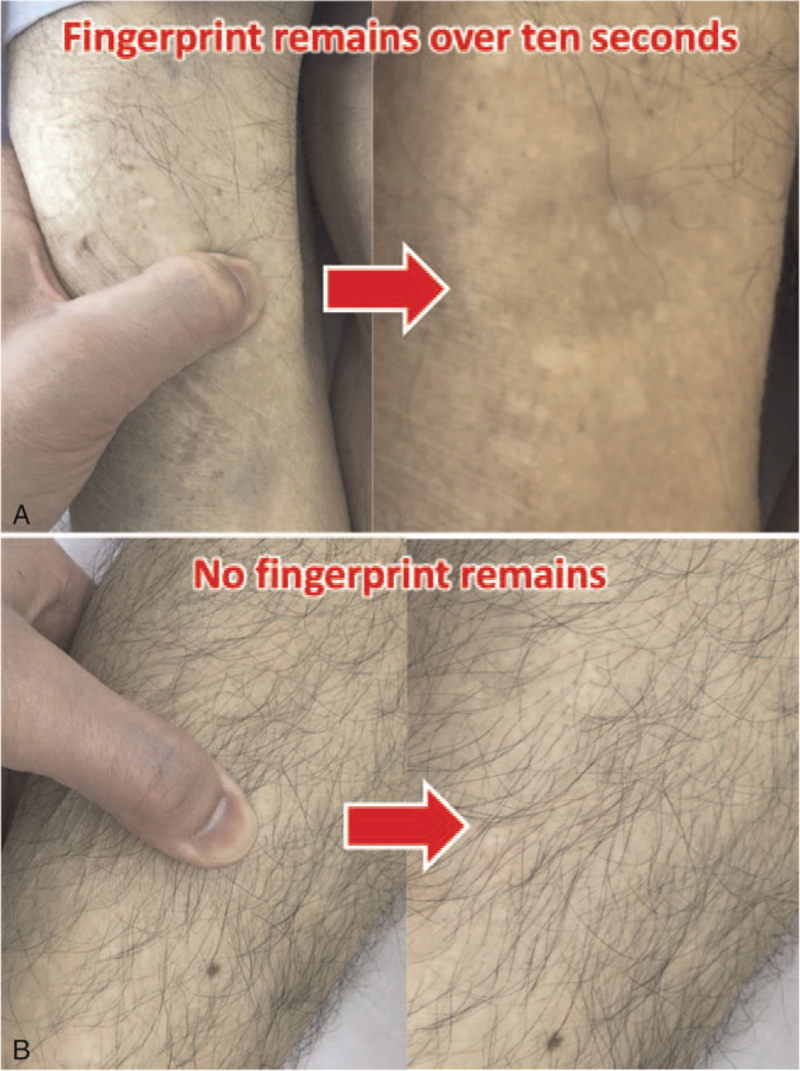
Criteria for determining the presence of edema. The presence/absence of edema was determined by the examiner pushing down strongly on the front surface of the tibia. The patients with remaining fingerprints for over ten seconds were assigned to the edema group, while the others were assigned to the no edema group. A) Edema group. B) No edema group.

We performed the study at the 18-bed medical-surgical ICU of Hitachi General Hospital, which admits about 1800 patients per year. The patients were enrolled between June 2018 and November 2018. The inclusion criteria were as follows: being aged >15 years, having tachycardic arrhythmia, and not being able to take oral medications because of swallowing dysfunction. Being subjected to arterial pressure monitoring was also a requirement for inclusion. Patients that met any of the following criteria were excluded: patients who had already been administered bisoprolol at their admission to the ICU and patients who still required catecholamine treatment. The latter criterion was employed because dermal drug absorption into the systemic circulation is impeded by impaired microperfusion at the application site.^[4]^ All candidates were examined for the presence/absence of edema, which was determined by the examiner pushing down strongly on the front surface of the tibia using their thumb, and then the patients on whom a fingerprint remained for over 10 seconds were assigned to the edema group, while the others were assigned to the no edema group (Fig. 1). We did not grade them by severity of edema in this study. These judgments were made by the physicians-in-charge, who were not blinded to the study design. Photographs of the patients’ tibiae were taken as evidence of the presence/absence of edema and preserved in our database. Since we have limited fund and had not yet obtained enough data to conduct a larger study at once, this study is planned as an exploratory investigation to assess pharmacokinetics variability caused by the presence of edema in patients. Thus, we enrolled only 6 patients in each group; that is, when one of the groups contained 6 patients, no further recruitment into that group was allowed.

A 4-mg bisoprolol dermal patch, Bisono Tape (Toa Eiyo Ltd., Tokyo, Japan), was pasted onto the precordium of each subject. Each subject's initial body temperature was documented since the skin penetration of drugs into the skin from dermal patches was reported to be thermosensitive.^[5]^ Even after a subject was included in the study, IV beta-blocker administration was possible without being excluded from the study if the patient's clinical course required it. Bisono Tape is usually changed every 24 hours. Therefore, each patient was observed for 24 hours after the application of the bisoprolol dermal patch, and their blood samples were to be collected via arterial line at 2, 4, 6, 8, 10, 12, 16, and 24 hours after the application of the patch. Every blood sample was quickly subjected to centrifugal separation (4°C, 3000 rpm, 15 minutes), before 2 mL of plasma was collected in a cryopreservation tube. These samples were preserved at −20°C. Finally, they were sent to a laboratory for examination. In the lab, bisoprolol was added to pooled blank human plasma to adjust 0.200 to 20.0 ng/mL calibration standards and 0.500, 2.00, and 16.0 ng/mL quality control samples. Metoprolol was used as an internal standard, and the calibration standards, quality control samples and assay samples were determined by the multiple reaction monitoring method of liquid chromatography–tandem mass spectrometry after solid phase extraction. Bisoprolol concentrations were calculated by substituting the obtained peak area ratios into the linear regression equation Y = aX + b.

According to the guideline of Food and Drug Administration, we considered that the comparison of total exposure between 2 groups should be done by AUC from time zero to time tau over a dosing interval at steady state (AUC_0–tau_), where tau is the length of the dosing interval.^[6]^ Thus, we set the primary endpoint of this study as the AUC_0–tau_. The secondary endpoints were the changes in HR and BP seen at 24 hours after the application of the patch. Early withdrawal was considered if a subject or their representative withdrew their consent or asked for the current therapy to be changed; if the physician-in-charge decided to discontinue the trial because of adverse events, such as worsening comorbidities or the new onset of illness; or for any other reasons.

Regarding the subjects’ baseline characteristics, continuous variables (expressed as mean and standard deviation values or median and IQR [1st–3rd] values) were compared between the 2 groups using the Mann–Whitney *U* test, whereas categorical variables (expressed as numbers and percentages) were compared with the chi-squared test. The blood concentrations of bisoprolol were compared using Mann–Whitney *U* test at each timepoint. The AUC, maximum concentration observed (C_max_) and also the reductions in HR and BP seen after 24 hours were compared between the groups using the Mann–Whitney *U* test. For all tests, *P*-values of <.05 were deemed significant.

This research was approved by the research ethics committee of Hitachi General Hospital (2018-40).

## 3 Results

### 3.1 Patients’ characteristics

None of the 12 patients left the trial after withdrawing their consent or suffering adverse events. The patients’ basic characteristics are shown in Table 1. There were no significant differences between the groups including with regard to renal function and body mass index, which have the greatest effect on blood bisoprolol concentrations. Although number of male patients appears to be slightly fewer in edema group, the difference was not statistically significant and might added no impact on the study result. The initial doses of landiolol did not differ significantly between the 2 groups. In addition, none of the patients’ landiolol doses changed during the observation period. There was no difference in the mean initial body temperature between the 2 groups. Also, as it was one of the exclusion criteria, there were no patients who were administered noradrenaline, which might inhibit drug absorption by decreasing drug microperfusion.

**Table 1:** Basic characteristics of the 2 groups.

	Edema group (n = 6)	No edema group (n = 6)	*P*-value
Age (median in years)	77.0 (73.5–85.3)	82.5 (73.5–85.3)	.629
Male, No. (%)	2 (33.3)	4 (66.7)	.564
BMI (kg/m^2^)	23.7 (19.2–26.0)	21.7 (18.7–24.0)	.298
APACHE 2	13.0 (9.0–17.3)	13.0 (10.8–17.3)	1.000
SOFA	8.0 (4.8–13.8)	8.5 (4.0–10.3)	.747
Atrial fibrillation, No. (%)	5 (83.3)	5 (83.3)	1.000
Initial HR (bpm)	98 (78–123)	98 (85–115)	.936
MAP (mm Hg)	80 (75–103)	87 (69–96)	.936
Body temperature (°C)	37.3 (36.8–38.4)	37.0 (36.4–37.2)	.257
BUN (mg/dL)	28.1 (19.7–42.7)	20.7 (13.1–41.8)	.521
Cre (mg/dL)	0.72 (0.58–1.72)	0.73 (0.59–1.67)	1.000
Lactate (mmol/L)	1.2 (0.8–1.5)	0.9 (0.7–1.1)	.259
Alive at discharge, No. (%)	4 (66.7)	6 (100)	.439
Admission period (days)	62.5 (31.3–94.8)	19.0 (11.8–48.3)	.174
Length of ICU stay (days)	9.0 (6.8–22.3)	8.0 (4.8–11.0)	.419
Dose of landiolol at start of bisoprolol patch use (μg/kg/min)	1.0 (0–4.8)	2.0 (0–5.9)	.797

Data are presented as the mean ± standard deviation or numbers (percentages). APACHE 2 = APACHE 2 disease classification score, BMI = body mass index, BUN = blood urea nitrogen level, Cre = creatinine level, HR = heart rate, MAP = mean arterial pressure, SOFA = Sequential Organ Failure Assessment score.

### 3.2 Outcomes

**Figure 2:**
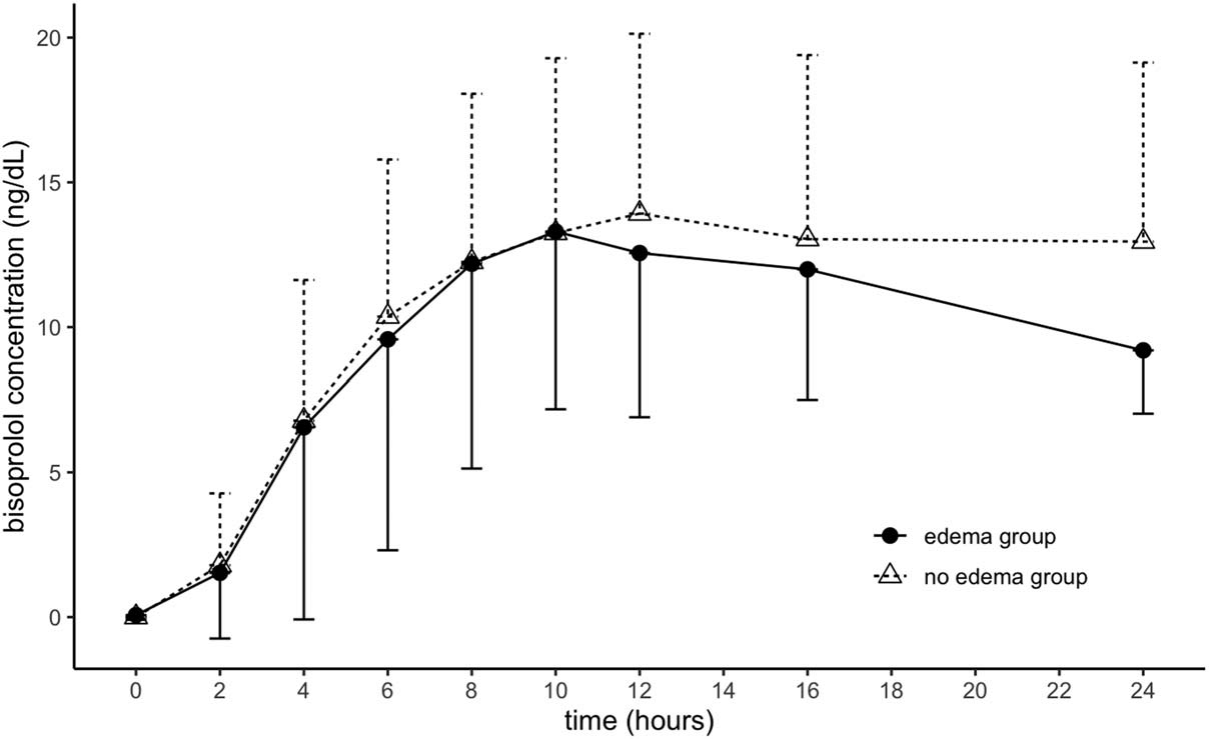
Time course of the blood bisoprolol concentration. The circles indicate the mean bisoprolol blood concentration of the edema group, whereas the triangles represent that of the no edema group. Data are presented as mean ± standard error values.

The mean blood bisoprolol concentration did not differ significantly between the 2 groups at 2, 4, 6, 8, 10, 12, 16, or 24 hours after the application of the patch (Fig. 2). The AUC_0–tau_ of 2 groups were 234.6 (131.5–319.1) ng hour/mL in the edema group and 290.6 (196.6–331.8) ng hour/mL in the no edema group (*P* = .689). Median C_max_ of the edema group were 13.3 (7.9–17.9) ng/mL, whereas that of no edema group were 17.0 (11.5–19.0) ng/mL (*P* = 0.6304). Median time of maximum concentration observed were 11.0 hours in edema group and 14.0 hours in no edema group. Thus, in terms of pharmacokinetics of the bisoprolol dermal patch, no significant difference was observed between 2 groups.

**Figure 3:**
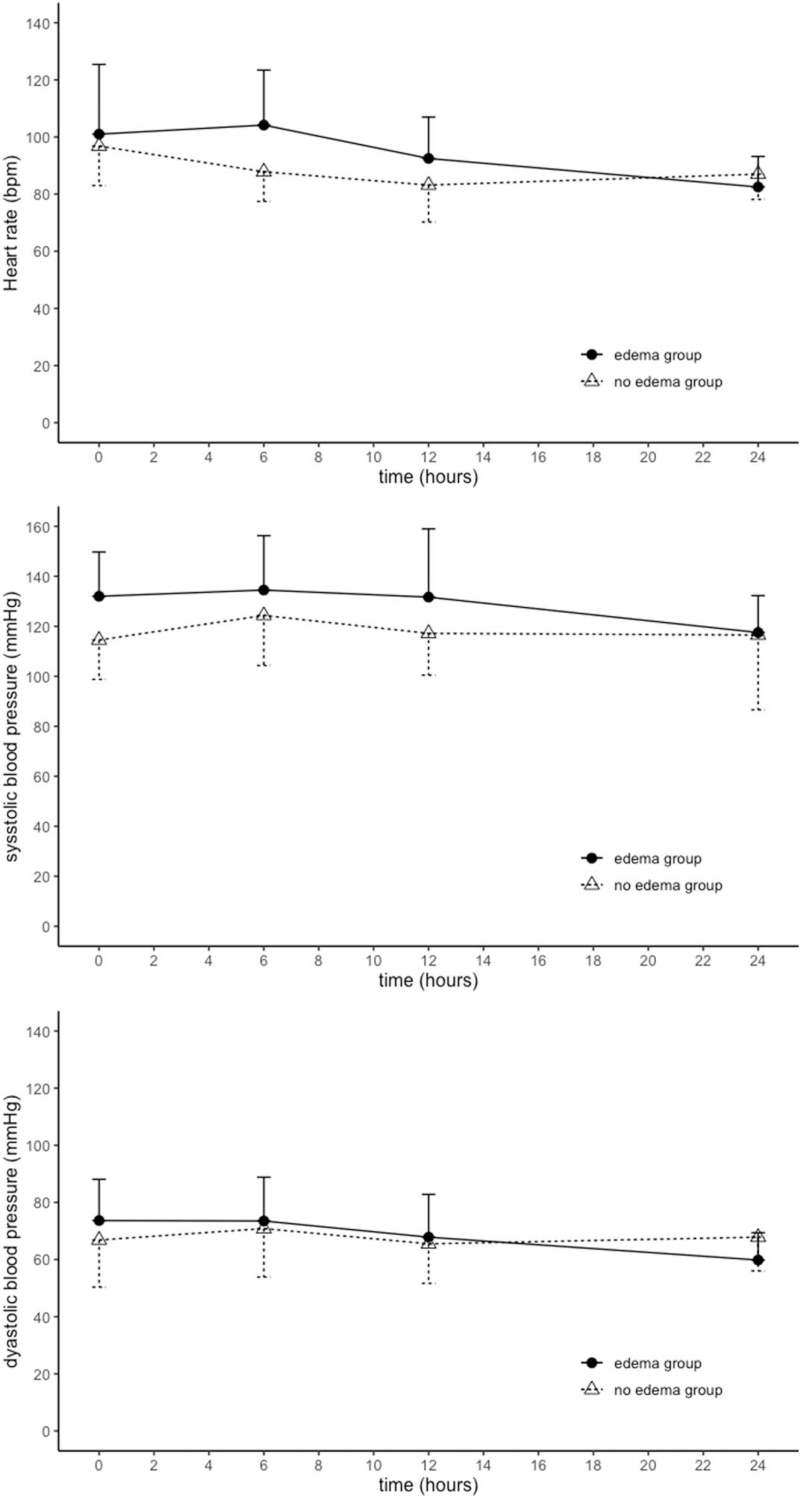
Secondary outcomes: the time–course of HR and BP. The circles indicate the mean HR or BP of the edema group, whereas the triangles represent that of the no edema group. Data are presented as mean ± standard error values. The reduction in HR seen after 24 hours did not differ significantly between the 2 groups (Mann–Whitney *U* test, *P* = .3768). BP = blood pressure, HR = heart rate.

The mean HR did not differ significantly between the 2 groups at 6, 12, or 24 hours after the application of the patch (Mann–Whitney *U* test, *P* = .2298, *P* = .3358, and *P* = .5752, respectively). Also, the reduction in HR seen after 24 hours did not differ significantly between the 2 groups (Mann–Whitney *U* test, *P* = .3768) (Fig. 3A). BP also did not differ significantly between the groups at any time point. BP was not significantly lower than the initial BP at any time point in either group (Fig. 3B and C).

## 4 Discussion

The main findings of the study were as follows. The AUC_0–tau_, mean C_max_, and median time of maximum concentration observed of bisoprolol which absorbed from dermal patch are not significantly different by the presence/absence of edema in lower extremities. In addition, the effects of the drug on HR and BP did not differ between the groups with and without edema. Our findings suggest that moderate to severe edema does not markedly affect the absorption or efficacy of bisoprolol in critically ill patients treated with bisoprolol dermal patches.

Although bisoprolol dermal patches are relatively new, dermal patches containing other drugs have been used for a while, and several studies about the pharmacokinetics of these products have been published. However, no previous study has focused on drug absorption in the presence of edema. Generally, the absorption of drugs from dermal patches is said to be improved when the amount of water in the skin is greater.^[7]^ The results of the present study do not contradict those of the latter study. Therefore, there seems to be no reason to avoid prescribing dermal patches to edematous patients.

The findings of this study also indicate that the HR-reducing effect of bisoprolol was preserved in the presence of edema, while no significant reduction in BP was observed in either group. According to a previous study of bisoprolol tablets, the HR reduction induced by the tablets was significantly greater in the 5 mg-treated group than in the 2.5 mg-treated group, whereas the effect on BP was quite mild and did not differ significantly between the 2 groups.^[8]^ Therefore, our findings might have been due to the drug characteristics of bisoprolol; that is, a small dose reduces HR dose-dependently and rarely produces a BP-reducing effect. Generally, the advantages of dermal patches are as follows: they avoid the first-pass effect of the liver; they result in the gradual elevation of the blood drug concentration, followed by the maintenance of an appropriate blood drug concentration; and they resolve the problem of adherence.^[9]^ Considering these advantages of dermal patches, we consider that it is reasonable and acceptable to use them as an alternative to oral bisoprolol drugs.

Our study had several limitations. First, the statistical power to detect differences was insufficient due to the relatively small sample size. Second, although we did not examine the concentration after 24 hours, it appears as if the difference of bisoprolol concentration between 2 groups to be larger after 24 hours from the patch's administration. Since bisoprolol patch is supposed to be changed every 24 hours, we think the pharmacokinetics after 24 hours may not be a great matter. Third, the presence/absence of edema was not determined via an objective method. Moreover, the severity of edema was not either evaluated or described. It may have influenced the result of the study. Finally, we did not evaluate edema in the precordium, where the patches were applied. Despite of these limitations, we still believe the topic of our study is important for clinicians. Thus, a randomized controlled trial with more examinee is warranted to truly evaluate the validity of our result.

## 5 Conclusion

In this study, after the application of a bisoprolol dermal patch, the pharmacokinetics of bisoprolol and its HR-reducing effects might not be affected by the presence of systemic edema in the lower extremities. Bisoprolol dermal patches may be a good treatment option, even for critically ill and edematous patients with tachyarrhythmia. However, further investigation is necessary to truly prove this result.

## Author contributions

All authors have contributed significantly; YT and KN conceived the study. All authors supervised the conduct of the study and data collection. HN managed the data, including figure drawing. YT drafted the article, and YG, TS, and KN contributed substantially to its revision. YT and KN take responsibility for the paper as a whole. All authors are in agreement with the content of the manuscript.

**Conceptualization:** Kensuke Nakamura.

**Data curation:** Hidehiko Nakano, Hiromu Naraba, Hideki Hashimoto.

**Formal analysis:** Hidehiko Nakano.

**Investigation:** Yuji Takahashi.

**Methodology:** Yuji Takahashi, Tomohiro Sonoo.

**Supervision:** Kensuke Nakamura.

**Writing – original draft:** Yuji Takahashi.

**Writing – review & editing:** Yuji Takahashi, Tomohiro Sonoo.
